# Nimbolide Inhibits SOD2 to Control Pancreatic Ductal Adenocarcinoma Growth and Metastasis

**DOI:** 10.3390/antiox12101791

**Published:** 2023-09-22

**Authors:** Tugba Mehmetoglu-Gurbuz, Rajkumar Lakshmanaswamy, Karla Perez, Mayra Sandoval, Casandra A. Jimenez, Jackelyn Rocha, Rachel Madeline Goldfarb, Courtney Perry, Alejandra Bencomo, Nishkala Neela, Jose A. Barragan, Raquel Sanchez, Risa Mia Swain, Ramadevi Subramani

**Affiliations:** 1 Center of Emphasis in Cancer Research, Department of Molecular and Translational Medicine, Paul L. Foster School of Medicine, Texas Tech University Health Sciences Center El Paso, El Paso, TX 79905, USA; 2 Francis Graduate School of Biomedical Sciences, Texas Tech University Health Sciences Center El Paso, El Paso, TX 79905, USA; 3 Paul L. Foster School of Medicine, Texas Tech University Health Sciences Center El Paso, El Paso, TX 79905, USA

**Keywords:** superoxide dismutase 2 (SOD2), reactive oxygen species (ROS), nimbolide, proliferation, metastasis, pancreatic cancer, pancreatic ductal adenocarcinoma, migration, invasion, epithelial-to-mesenchymal transition

## Abstract

Reactive oxygen species are frequently associated with various cancers including pancreatic ductal adenocarcinomas (PDACs). Superoxide dismutase 2 (SOD2) is an enzyme that plays an important role in reactive oxygen species (ROS) signaling. Investigating the molecular function and biological functions of SOD2 can help us develop new therapeutic options and uncover new biomarkers for PDAC diagnosis and prognosis. Here, we show that nimbolide (NB), a triterpene limonoid, effectively blocks the growth and metastasis of PDACs by suppressing the expression and activity of SOD2. To identify the role of SOD2 in NB-induced anticancer activity, we used RNA interference to silence and plasmid transfection to overexpress it. Silencing SOD2 significantly reduced the growth and metastatic characteristics like epithelial-to-mesenchymal transition, invasion, migration, and colony-forming capabilities of PDACs, and NB treatment further reduced these characteristics. Conversely, the overexpression of SOD2 enhanced these metastatic characteristics. ROS signaling has a strong feedback mechanism with the PI3K/Akt signaling pathway, which could be mediated through SOD2. Finally, NB treatment to SOD2-overexpressing PDAC xenografts resulted in significant inhibition of tumor growth and metastasis. Overall, this work suggests that NB, a natural and safe phytochemical that silences SOD2 to induce high levels of ROS generation, results in increased apoptosis and reduced growth and progression of PDACs. The role of SOD2 in regulating NB-induced ROS generation presents itself as a therapeutic option for PDACs.

## 1. Introduction

Pancreatic cancer has one of the worst prognoses [1] among all cancers due to its high ability to micrometastasize [2]. While it accounts for only 3% of the total number of cancers diagnosed, it accounts for 7% of all cancer-related deaths [3,4]. The most common mutation, seen in 90–95% of pancreatic cancer cases, is the KRAS2 oncogene activation [5]. A mutation in the K-Ras gene leads to an imbalance in the redox status, leading to the accumulation of superoxide radicals and causing tumorigenic behavior of pancreatic cells [6]. Altered ROS levels are well-known indicators in many types of cancer, including pancreatic cancer [6,7]. ROS is a double-edged sword; it is required for regulating signaling pathways [8] involved in cell proliferation, differentiation, and survival [9], as well as cellular functions [8]. Slightly increased levels of ROS create a beneficial environment for cancer cell proliferation [10]. By contrast, higher levels of ROS could lead to cell death [11]. 

SOD enzymes control the levels of a variety of ROS. Three highly compartmentalized types of SOD are expressed in human cells. SOD1 (Cu/Zn SOD) is a 32 kDa homodimer, primarily located in the cytoplasm, nucleus, peroxisomes, and intermembrane space of the mitochondria [12]. SOD2 (MnSOD) is a 96 kDa homotetramer, which is mainly located in the mitochondrial matrix. Finally, SOD3 (ecSOD) is a 135 kDa homotetrameric secretory glycoprotein found in the extracellular matrix, cell surface, and extracellular fluids. This compartmentalization is required for the control of ROS signaling and to effectively counteract oxidative stress.

Effective treatments for PDACs remain a challenge. Gemcitabine is a standard chemotherapeutic drug that has been used in the treatment of PDACs. Its efficacy has been demonstrated by inhibiting the replication of cancer cells [13]. Gemcitabine has mostly been used as a monotherapy in clinical applications. Gemcitabine has also been combined with other treatments to increase efficacy. Gemcitabine plus paclitaxel or gemcitabine plus platinum agents may be more effective but still face heavy resistance [13]. Hence, exploring novel therapeutic modalities allows for enhancing treatment outcomes for patients with PDACs.

Nimbolide (5,7,4′-trihydroxy-3′5′-diprenyl flavanone, C_27_H_30_O_7_) (NB), a tetranortriterpenoid extracted from the leaves and flowers of *Azadirachta indica*, is known from its many medicinal features such as its antihistamine [14], antipyretic [15], antifungal, antiarthritic [16], anti-inflammatory [17], antiviral [18], antihyperglycemic [19], antifeedant [20], antimalarial [21], antimicrobial [22], anti-HIV [23], pesticidal [24], spermicidal [25], and anticancer properties [26,27]. Earlier, we demonstrated NB as an effective anticancer agent against pancreatic ductal adenocarcinomas. We also observed that NB administration resulted in increased generation and accumulation of ROS, leading to the death of pancreatic cancer cells. Nimbolide was also effective in inhibiting the metastatic and invasive nature of pancreatic cancer cells [28]. Although the anticancer effects of NB are established, the underlying mechanisms are still not well understood.

In the present study, we uncover a novel mechanism through which NB induces ROS generation, thereby inhibiting the growth and progression of pancreatic ductal adenocarcinomas. Our data demonstrate that alterations in SOD2 expression and activity levels play a major role in regulating redox signaling, which influences the PI3K/Akt signaling pathway. As ROS-induced alterations in the survival signaling pathways lead to the inhibition of pancreatic ductal adenocarcinomas, an in-depth understanding of the interactions between these two aspects is vital for the future design and development of mechanism-based therapeutics for pancreatic ductal adenocarcinoma.

## 2. Materials and Methods

### 2.1. Cell Lines

Pancreatic duct normal epithelial cell line (hTERT-HPNE) and pancreatic ductal adenocarcinoma cell lines such as HPAC, AsPC-1, Capan-1, Capan-2, PANC-1, MIAPaCa-2, and BxPC-3 were procured from the American Type Culture Collection (ATCC-CRL-2119, Manassas, VA, USA) and maintained at 37 °C in a humidified carbon dioxide incubator set to 5%.

### 2.2. Immunohistochemistry

Immunohistochemistry was performed as described earlier [28,29]. In brief, a human PDAC tissue microarray (PA484, US Biomax, Rockville, MD, USA) and HPAC cell line xenograft tissues were used to assess the expression of SOD2 (13141), pAkt (4060), BAX (2772), Snail (3879), cleaved caspase 9 (9509), pmTOR (5536), and E-cadherin (3195) proteins (Cell signaling Technology Inc., Danvers, MA, USA). The experimental conditions and detailed protocols are described in the Supplementary Materials.

### 2.3. Determining SOD2-Induced Alteration in Tumor Using In Silico Analysis

In silico analysis was performed to compare the expression of SOD2 in different tumors as well as the different tumor grades of pancreatic cancer. The graph depicting SOD2 expression across different tumor grades was generated using the UALCAN website (https://ualcan.path.uab.edu/cgi-bin/ualcan-res.pl, accessed on 10 January 2022). UALCAN’s analysis tools allow for the visualization of SOD2 expression patterns in relation to varying tumor grades. The heat-map representation of SOD1, SOD2, SOD3, and CAT expression in different cancer tissues, including pancreatic cancer, was generated using online databases. The plots comparing normal, tumor, and metastatic expression of SOD2 were generated using the TNMplot analysis platform (https://tnmplot.com/analysis/, accessed on 10 January 2022).

### 2.4. Survival Analysis

To study the impact of individual genes on overall survival probability and recurrence-free survival, we used the KM plotter (http://kmplot.com/, accessed on 10 January 2022) using mRNA RNA seq data.

### 2.5. Immunofluorescence Analysis

Immunofluorescence was performed as described earlier [30,31]. In brief, HPAC cells were transfected with either SOD2 siRNA (siSOD2) or SOD2 plasmid DNA (ovSOD2). The expression of SOD2, p-Akt, pmTOR, E-cadherin, Slug, and BAX proteins were assessed. The experimental conditions and detailed protocols are described in the Supplementary Materials.

### 2.6. Immunoblotting

The expression patterns of key signaling molecules were analyzed via immunoblot analysis as described earlier [28,32]. In brief, the effect of NB and SOD2 on the following proteins was assessed: SOD2, pAkt, pmTOR, pP70 S6K, pPI3K, Vimentin, E-cadherin, cleaved caspase 8, cleaved caspase 9, BAX, N-cadherin, and Snail, purchased from Cell Signaling Technology (Danvers, MA, USA); ß-actin (A1978), purchased from Sigma-Aldrich (St. Louis, MO, USA); Zeb-1 and pERK, purchased from Santa Cruz Biotechnology (Santa Cruz, CA, USA); and Twist, purchased from Abcam (Cambridge, UK). The experimental conditions and detailed protocols are described in the Supplementary Materials.

### 2.7. Silencing SOD2 in Pancreatic Cell Line

Three different SOD2 siRNA subtypes were obtained from OriGene (Cat# SR321855, Rockville, MD, USA) with different sequences (see Supplementary Materials) were used. Mirus bio TransIT siQUEST (Cat#MIR2110, Marietta, GA, USA) transfection reagent was used for transfection. A scramble siRNA sequence was used as a negative control for non-sequence-specific effects. Among the three subtypes of SOD2 siRNA, siRNA ‘A’ showed maximum efficacy in silencing SOD2. Following the 48 h transfection, cells were processed for further experiments.

### 2.8. Overexpression of SOD2 in Pancreatic Cell Line

A plasmid overexpressing SOD2 (ovSOD2) gene was obtained from OriGene (CW303931, Rockville, MD, USA). HPAC cells were seeded in 6-well plates at a density of 5 × 10^5^ cells/well and transfected with SOD2 plasmid DNA at a concentration of 10 µg/µL using a Mirus 2020 (Cat#MIR5400, Marietta, GA, USA) transfection reagent. An empty plasmid vector was used as a control. After 48 h of transfection, green fluorescence analysis was performed using a FLoidCell Imaging Station (Life Technologies, Thermo Fisher Scientific Inc., Waltham, MA, USA) to determine the efficacy of transfection for further analysis.

### 2.9. SOD2 Zymography

SOD2 activity was analyzed using zymography. Briefly, 100 µg protein from SOD2-transfected HPAC cells in the presence and absence of NB were loaded on non-denaturing acrylamide gels, followed by electrophoresis. SOD2 activity was visualized by observing the inhibition of nitroblue tetrazolium using the nitroblue tetrazolium dye reduction test.

### 2.10. Cell Proliferation Using MTS Assay

HPAC cells were seeded in a 96-well plate at a density of 5 × 10^3^ cells per well in triplicates. Cell proliferation was determined by using an MTS assay. HPAC cells were exposed to NB at varying doses for 24 h. According to the manufacturer’s protocol, an MTS reagent was added into each well and incubated for 4 h. Optical density was measured using a BMG Labtech CLARIOstar microplate reader at 490 nm absorbance.

### 2.11. ROS Generation Assay

Intracellular ROS generation was assessed using an OxiSelect Intracellular ROS assay kit purchased from Cell BioLabs Inc., San Diego, CA, USA (STA-342). HPAC cells were seeded in a 96-well plate at a density of 2 × 10^4^ cells per well. After 24 h, cells were treated with Wortmannin, LY294002, and NAC for 24 h or transfected with SOD2 siRNA, or SOD2 pcDNA for 48 h. Then, cells were washed with PBS three times and incubated with 2′,7′-dichlorodihydrofluorescin diacetate (DCFH-DA) for 45 min at 37 °C in the dark. DCFH-DA was then removed, and cells were treated with 5 µM of NB. After 20 min, 1× cell lysis buffer was added and mixed thoroughly for 5 min. Cell lysate from each replicate was transferred into a black bottom fluorimetric 96-well plate for fluorescence measurement using a BMG LabTech fluorescence plate reader at 480 nm excitation and 530 nm emission. Data are expressed as the amount of ROS relative to non-transfected and untreated control groups.

### 2.12. Transwell Migration and Matrigel Invasion Assay

Migration and invasion assays were performed as described earlier [28,33]. The experimental conditions and detailed protocols are described in the Supplementary Materials.

### 2.13. Soft Agar Colony Formation Assay

siSOD2 or ovSOD2-transfected HPAC cells were treated with 5 µM of NB. After 24 h, cells were reseeded at a density of 1.5 × 10^5^ cells into 60 mm Petri dishes with a top layer of 0.7% agarose (Agarose Ultrapure, Invitrogen,16500-100, Thermo Fisher Scientific, MA, USA) and a bottom layer of 1% agar (Agar Difco Nutrient, Becton Dickinson, DF001-17-0). Non-transfected and untreated cells were used as controls. Cells were maintained for up to 45 days and then stained with 0.2% crystal violet. Images of cell colonies were captured using a Nikon SMZ 1500 microscope at 10 and 40× magnifications.

### 2.14. Wound-Healing Assay

SOD2-silenced and SOD2-overexpressing HPAC cells with or without NB treatment were seeded in 6-well plates at a density of 1.2 × 10^6^ cells/well and maintained for 96 h. A scratch was created with a sterile pipette tip once the cells reached monolayer confluency. The distance migrated by the cells was captured and calculated at 2 h intervals for 96 h using the Nikon Biostation CT. NIS-Element AR software was used for analysis.

### 2.15. Apoptosis Analysis and Flow Cytometry

Apoptosis was analyzed using Annexin V-FITC Apoptosis Detection Kit (556547) according to the manufacturer’s instructions (BD Biosciences, San Diego, CA, USA). Cells were seeded in 6-well plates at a density of 5 × 10^5^ cells/well and were treated with 5 μM NB for 24 h or transfected with SOD2 plasmid or siRNA for 48 h. Apoptosis was studied using a FACS Accuri C6 flow cytometer (San Jose, CA, USA).

### 2.16. Xenograft Model

All animal studies were approved by the Institutional Animal Care and Use Committee of the Texas Tech University Health Sciences Center, El Paso. SOD2-overexpressing HPAC cells were implanted subcutaneously in the right and left flanks (1.0 × 10^6^ cells/flank) of six-week-old athymic nude mice (JAX Lab) (*n* = 8). Their respective parental cells were transplanted subcutaneously and used as controls. Once tumors became palpable (~100 mm^3^), NB (5 mg/kg body weight; 5 days/week for 1 month) was administered via intraperitoneal injections to each mouse in the treatment group. Dimethyl sulfoxide was administered in vehicle control. Tumor volume and body weight were recorded weekly. Tumor volume was compared between control and treatment groups using the formula 4/3π × r1^2^ × r2, where r1 is the minor radius, and r2 is the major radius. The mice were euthanized to collect tumors, as well as tissues from various organs (brain, lung, and liver) for further analysis. A portion of each of the tissues was fixed in 10% formalin for histopathological analysis and immunohistochemistry. The remainder of the tissue sample was flash-frozen in liquid nitrogen to be used for molecular analysis [29,31].

### 2.17. Statistical Analysis

GraphPad Prism version 6.01 software (GraphPad Software Inc., Boston, MA, USA) was used to perform all statistical analysis. To study the difference in effect between groups, the sample size was determined using Power analysis. Statistical significance was calculated using two-tailed unpaired *t*-tests on two groups. A value of *p* ≤ 0.05 showed significance. All in vitro experiments were replicated at least 3 times and analyzed. The data are expressed as means ± standard deviations.

## 3. Results

### 3.1. Abundant Expression of Superoxide Dismutase-2 (SOD2) in PDACs

**Figure 1:**
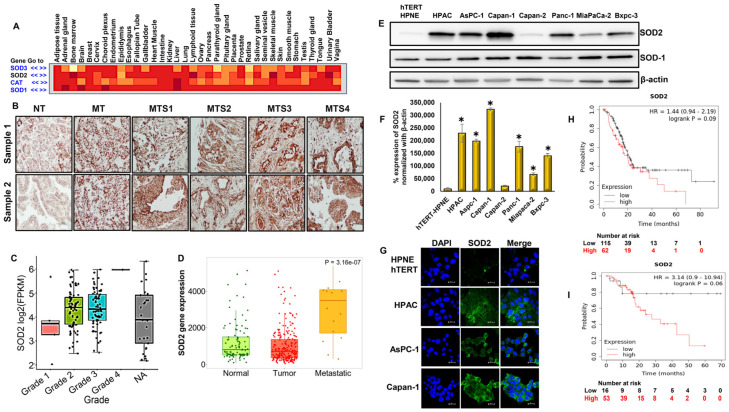
**SOD2 overexpression is associated with poor survival in pancreas adenocarcinoma:** (**A**) heat-map representation of SOD1, SOD2, SOD3, and CAT expression in different cancer tissue including pancreatic cancer; (**B**) immunohistochemistry analysis of SOD2 expression in normal pancreas and different stages of human pancreatic ductal adenocarcinoma tumor tissues; SOD2-positive cells indicated by brown staining at 40× magnification (NT—normal tissue, MT—malignant tissue, MTS1—malignant tissue stage 1; MTS2—malignant tissue stage 2, MTS3—malignant tissue stage 3, MTS4—malignant tissue stage 4); (**C**) expression of SOD2 in pancreatic adenocarcinoma based on tumor grade using UALCAN; (**D**) SOD2 expression in normal, tumor, and metastatic pancreatic adenocarcinoma cells were compared using TNMplot analysis; (**E**) Western blot and (**F**) densitometric analysis of SOD2 expression in a panel of pancreatic cancer cell lines and normal pancreas cell line; Each bar represents the mean ± SEM of three separate experiments, * *p* < 0.05; (**G**) immunofluorescence analysis of SOD2 expression in pancreatic ductal adenocarcinoma cells compared with normal pancreas cells at 100× magnification via confocal microscopy; (**H**) Kaplan–Meier plot for overall survival; (**I**) recurrence-free survival (months) for SOD2 expression.

SOD2 expression is higher than SOD1, SOD3, and CAT in pancreatic cancers (Figure 1A). Immunohistochemical analysis was conducted to explore the pathophysiological relationship between PDAC stage and SOD2 levels, using a pancreatic cancer tissue microarray, which consisted of different grades of pancreatic cancer and also normal pancreatic tissues. Our results indicate that SOD2 expression was higher in cancers than in normal pancreas (Figure 1B). Furthermore, SOD2 expression increased proportionately with the increasing grade of pancreatic cancers (Figure 1C), demonstrating a positive correlation between pancreatic cancer progression and SOD2 expression. Moreover, the SOD2 gene was expressed at higher levels in metastatic sites than in primary tumor and normal tissues (Figure 1D).

Next, we assessed the expression levels of SOD2 in a panel of PDAC cell lines using Western blot and immunofluorescence analysis. We detected higher expression levels of SOD2 in all PDAC cell lines (HPAC, AsPC-1, Capan-1, PANC-1, MiaPaCa-2, and BxPC-3) except for Capan-2 cells compared with the non-cancerous pancreatic cell line, hTERT-HPNE. Interestingly, SOD1 levels in all cell lines were comparable (Figure 1E). In particular, aggressive pancreatic cancer cell lines such as HPAC, AsPC-1, Capan-1, and PANC-1 displayed the highest levels of SOD2 (Figure 1F,G). HPAC cells were selected for further experiments to study the role of SOD2 in NB’s anticancer effect against pancreatic cancer.

In silico analysis was performed to identify the prognostic value of SOD2 in pancreatic cancer patients using the Kaplan–Meier plot. The Kaplan–Meier survival curve data demonstrated that elevated expression levels of SOD2 are associated with reduced overall survival time (months, log-rank, *p* = 0.09) and recurrence-free survival time (months, log-rank, *p* = 0.06) (Figure 1H,I).

### 3.2. SOD2 Influences Cell Survival and Proliferation through PI3K/Akt/mTOR Pathway in Pancreatic Cancer

**Figure 2:**
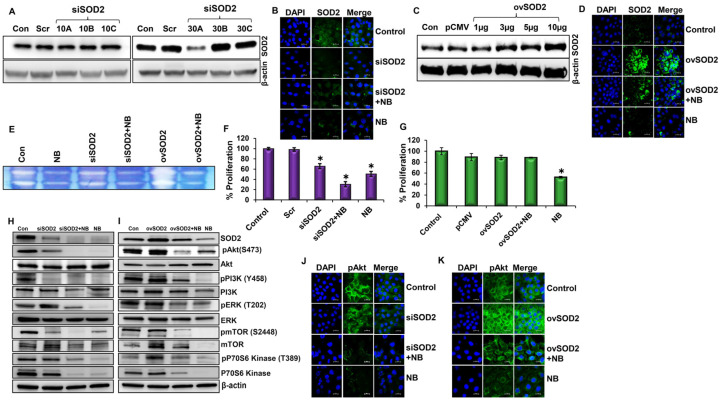
**Nimbolide inhibits cell proliferation through SOD2 inhibition:** (**A**) Western blot analysis of SOD2 protein expression in SOD2-silenced HPAC pancreatic cancer cells using three different predesigned siRNAs at 10 or 30 nM for 48 h; (**B**) immunofluorescence analysis of SOD2 expression in SOD2-silenced (siSOD2) HPAC cells alone or in combination with 5 µM of NB at 100×magnification via confocal microscopy; (**C**) Western blot analysis of SOD2 expression in SOD2-overexpressed (ovSOD2) HPAC cells at 1, 3, 5, and 10 µg; (**D**) immunofluorescence analysis of SOD2 in SOD2-overexpressed HPAC cells with or without 5 µM of NB treatment with 100× magnification; (**E**) zymography analysis of SOD2 enzyme activity in siSOD2 and ovSOD2 cells in the presence or absence of 5 µM of NB. HPAC cell proliferation was studied using an MTS assay kit; (**F**) siSOD2 and (**G**) ovSOD2 with or without 5 µM of NB treatment; (**H**) Western blot analysis of proliferation markers in siSOD2 and (**I**) ovSOD2 HPAC cells with or without 5 µM of NB treatment. Immunofluorescence analysis of pAkt in (**J**) siSOD2 and (**K**) ovSOD2-transfected HPAC cells with or without 5 µM of NB with 100× magnification. Each bar represents the mean ± SEM of three separate experiments, * *p* < 0.05.

In this study, we aimed to understand the role of SOD2 in pancreatic cancer. Therefore, we knocked down the expression of SOD2 in HPAC cells by utilizing predesigned siRNAs at concentrations of 10 nm and 30 nm using subtypes ‘A’, ‘B’, and ‘C’. Scrambled siRNA, which does not target any gene, was used as an internal control. Western blot analysis demonstrated the effective silencing of SOD2 in HPAC cells. Specifically, SOD2 expression levels were most efficiently knocked down by siRNA subtype ‘A’ at a concentration of 30 nM (Figure 2A and Figure S1A,B). Immunofluorescence results further confirmed the silencing expression of SOD2 (Figure 2B). Therefore, this siRNA subtype and concentration were selected for all subsequent studies.

To further understand the role of SOD2 in NB-induced anticancer activity, it was overexpressed in HPAC cells with various concentrations (1 µg, 3 µg, 5 µg, and 10 µg) of SOD2 plasmid DNA to identify the optimal dose. SOD2 plasmid was tagged with green fluorescence protein (GFP) and a target gene transfected with the cytomegalovirus (CMV) promoter. Cells transfected with 10 µg of SOD2 plasmid DNA exhibited the highest expression of SOD2 (Figure 2C and Figure S1C). The SOD2 overexpression was confirmed using fluorescence microscopy (Figure S1D). These results were further validated with immunofluorescence microscopy (Figure 2D).

Zymography was used to assess the SOD2 enzyme activity in NB-treated SOD2-altered cells using native gel electrophoresis. As expected, silencing SOD2 decreased SOD2 activity, and overexpressing SOD2 increased SOD2 activity. As noticed in Figure 2B,D, treatment with NB decreased SOD2 activity in both SOD2-silenced and SOD2-overexpressed cells compared to their respective controls (Figure 2E).

In our previous study, we demonstrated that NB was effective in inhibiting pancreatic cancer growth by inducing excessive ROS [28]. However, the molecular mechanism is not well established. In this study, we treated SOD2-silenced cells with an IC_50_ dose (5 µM) of NB (NB). This significantly decreased the proliferation rate of SOD2-silenced HPAC cells compared with scrambled siRNA-transfected controls. Nimbolide treatment in SOD2-silenced HPAC cells inhibited proliferation significantly better than the NB-alone group. These data demonstrate that NB-induced ROS inhibits the proliferation of PDAC cells (Figure 2F and Figure S1E). SOD2 overexpression (ovSOD2) increased the proliferation rate of HPAC cells compared with the NB-treated group. However, in contrast to NB treatment alone, the ovSOD2 cells treated with NB were comparable with the control, which indicates that NB-induced ROS is key to regulating the proliferation of PDAC cells (Figure 2G and Figure S1F).

We performed the PhosphoExplorer protein antibody microarray, and interestingly, the data revealed that PI3K/Akt and ROS signaling are the two most affected pathways by NB in pancreatic cancer cells (Figure S2A,B). The microarray data further demonstrated that NB reduced the expression of pAkt, pBAX, pPTEN, and pVEGF, which could have resulted in inhibiting PDAC growth and progression (Figure S2C–F).

Next, we investigated the effect of NB-induced ROS on the survival and proliferation of pancreatic cancer cells. Silencing SOD2 in HPAC cells significantly decreased the expression levels of the active forms of PI3K, Akt, ERK1/2, and mTOR, as well as both the total and active forms of p70s6k. NB-treated SOD2-silenced HPAC cells had reduced levels of pPI3K, pAkt, pERK, pp70s6k, and pmTOR compared with untreated SOD2-silenced cells (Figure 2H). By contrast, SOD2 overexpression increased the active forms of Akt, PI3K, ERK, mTOR, and p70S6K compared with NB-treated SOD2-overexpressing cells (Figure 2I). This indicated that NB increased ROS by decreasing the expression levels of SOD2 to control PDAC proliferation via PI3K/Akt signaling. Immunofluorescence imaging also verified the reduced expression of proliferative markers pAkt and pmTOR in response to the suppression of SOD2, which was further reduced by NB treatment (Figure 2J and Figure S3A). On the other hand, the overexpression of SOD2 increased pAkt and pmTOR, while NB treatment brought down pAkt expression to control levels (Figure 2K). A similar trend of expression was also observed for pmTOR in the various groups (Figure S3B).

### 3.3. Inhibition of PI3K/Akt Signaling by NB Is Caused by the Induction of ROS via SOD2 Silencing

**Figure 3:**
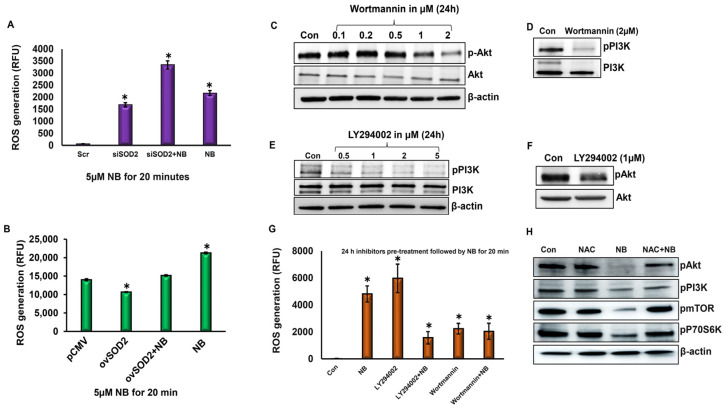
**NB-induced ROS generation via SOD2 inhibits PI3K/Akt signaling.** Mitochondrial ROS levels were assessed in (**A**) SOD2-silenced and (**B**) SOD2-overexpressed HPAC pancreatic cancer cells with or without 5 µM of NB treatment for 20 min using a fluorescence plate reader; (**C**) HPAC pancreatic cancer cells were treated with P13K/Akt inhibitor Wortmannin (0.1–2 µM) for 24 h, and pAkt and Akt expression levels were assessed using western blot; (**D**) expression levels of pPI3K and PI3K levels were tested in Wortmannin (2 µM)-treated HPAC cells for 24 h; (**E**) pPI3K and PI3K protein expression levels were determined on HPAC pancreatic cancer cells treated with P13K/Akt inhibitor LY294002 (0.5–5 µM) for 24 h; (**F**) expression levels of pAkt and Akt levels were tested in LY294002 (1 µM)-treated HPAC cells for 24 h; (**G**) ROS production was assessed in HPAC pancreatic cancer cells pretreated with Akt/PI3K inhibitors (1 μM of LY294002 or 2 μM Wortmannin) for 24 h, followed by NB treatment 20 min via flow cytometry; (**H**) cell proliferation/survival markers were assessed in HPAC cells in the presence of ROS inhibitor N-acetyl-L-cysteine (NAC) with or without NB treatment. Each bar represents the mean ± SEM of three separate experiments, * *p* < 0.05.

Earlier, a time-course experiment using 5 µM of NB demonstrated that ROS generation occurs early and remains stable up to 6 h [28]. Silencing SOD2 increased ROS level by nearly 1.5-fold compared with the control group. An additive effect was observed in the combination group (siSOD2 + NB) (Figure 3A). By contrast, the overexpression of SOD2 lowered the basal ROS levels in contrast to the untreated control group. Interestingly, NB treatment increased ROS levels even in SOD2-overexpressing cells (Figure 3B). Thus, even with enhanced antioxidant mechanisms, NB is able to alter mitochondrial ROS levels in pancreatic cancer cells.

To ascertain if PI3K/Akt signaling is upstream of ROS production, HPAC pancreatic cancer cells were pretreated with PI3K/Akt inhibitors LY294002 (LY; 1 μM) or Wortmannin (WM; 2 μM), followed by NB treatment. In order to identify the most effective doses for inhibiting the constitutional activation of PI3K and Akt, doses ranging from 0.1 to 2 µM were tested for WM, while 0.5–5 µM concentrations were tested for LY. Activated PI3K/Akt expression levels were significantly decreased with 2 µM WM (Figure 3C,D) and 1 µM LY (Figure 3E,F), which was selected for further studies.

To investigate if the inhibition of PI3K/Akt signaling by NB regulates ROS generation, we measured ROS generation after 24 h of pretreatment with LY or WM, followed by 20 min treatment with NB. We determined that the LY increased ROS level similar to the NB treatment group compared with control cells. The LY + NB combination group had higher levels of ROS than controls but had lower levels than LY- or NB-alone groups. WM alone and in combination with NB also increased ROS generation compared with the control. As expected, the ROS level was higher in NB-treated cells than in the control group (Figure 3G). These data suggest that there is a strong possibility for feedback regulation between ROS and PI3K/Akt signaling in NB-treated pancreatic cancer cells. 

To further confirm the effect of NB-induced ROS generation on the PI3K/Akt signaling in pancreatic cancer cells, ROS generation was inhibited by a pharmacological inhibitor, N-acetyl-l-cysteine (NAC). As expected, we observed the inhibitory effect of NB on pAkt, pPI3K, and pmTOR, and pp70S6K levels. NB’s inhibitory effects on PI3K/Akt signaling were restored with NAC pretreatment, bringing the levels similar to those of the control group (Figure 3H). The inhibition of ROS by NAC activates PI3K/Akt signaling even in the presence of NB, demonstrating that NB inhibits pancreatic cancer through ROS generation.

### 3.4. Differential SOD2 Expression Levels Influence Migration and Invasion Capabilities

**Figure 4:**
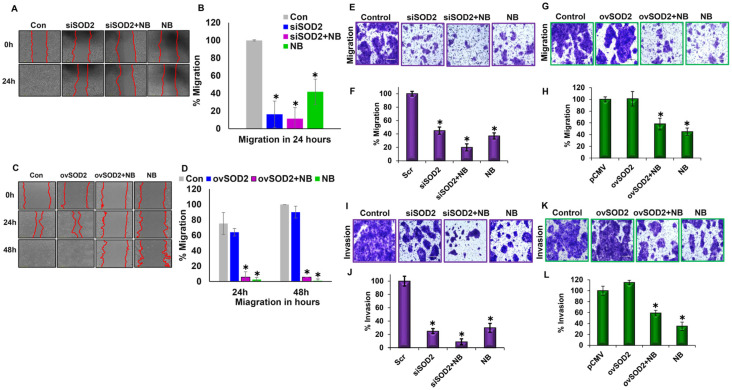
**Differential SOD2 expression levels influence migration and invasion capabilities:** (**A**) a wound-healing assay was performed at 0 and 24 h in SOD2-silenced HPAC cells treated with or without 3 µM of NB; (**B**) quantification of migration in SOD2-silenced PDAC cells after NB treatment was performed with NIS-Element AR software; (**C**) a wound-healing assay was performed at 0, 24, and 48 h in SOD2-overexpressed HPAC cells treated with or without 3 µM of NB; (**D**) quantification of migration in SOD2-overexpressed PDAC cells after NB treatment was carried out with NIS-Element AR software; (**E**) HPAC cell migration was analyzed in SOD2-silenced treated with or without NB using a transwell migration assay; (**F**) the percentage of migration was quantified with the control cell serving as the baseline; (**G**) HPAC cell migration was assessed in SOD2-overexpressed treated with or without NB using a transwell migration assay; (**H**) quantification of colonies in the overexpressed group was performed with the control cell serving as the baseline; (**I**) a Matrigel invasion assay was performed to assess the invasion of SOD2-silenced PDAC cells with or without NB; (**J**) the percentage of invaded SOD2-silenced PDAC cells was calculated with the control cell serving as the baseline; (**K**) an invasion assay was performed to assess the invaded cells in the SOD2-overexpressed group treated with or without NB; (**L**) the percentage of invaded cells in SOD2-overexpressed groups was calculated with the control cell serving as the baseline. Each bar represents the mean ± SEM of three separate experiments, * *p* < 0.05. A paired *t*-test was performed to determine statistical significance.

The migratory and invasive capacities of cancer cells are crucial for cancer progression and metastasis. The migratory abilities of HPAC cells were evaluated using a scratch assay. SOD2 silencing, NB-alone treatment, and the combination significantly inhibited migration compared with controls (Figure 4A,B), whereas SOD2-overexpressing cells had similar migratory capacity as control cells, but NB was highly efficient in decreasing migration even in SOD2-overexpressing cells (Figure 4C,D). To confirm these findings, a transwell migration assay was performed. We observed a similar effect of altering the expression levels of SOD2 and NB treatment on HPAC cell migratory capacity (Figure 4E–H). To determine the invasive capabilities of HPAC cells, Matrigel-coated Boyden chambers were used. Our data demonstrated that NB, siSOD2, and siSOD2 plus NB treatment significantly inhibited the invasion of HPAC cells compared with control cells (Figure 4I,J), while the overexpression of SOD2 reduced the inhibitory effect of NB on PDAC invasive capacity (Figure 4K,L).

### 3.5. SOD2 Regulates Anchorage-Independent Growth of Pancreatic Cancer Cells

**Figure 5:**
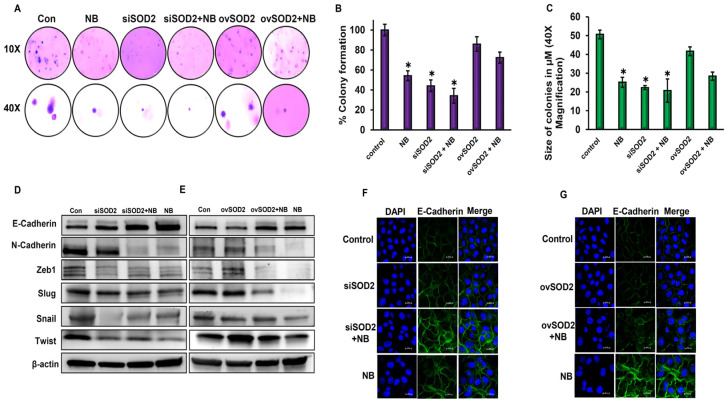
**Altered SOD2 expression modulates colony formation and EMT in response to nimbolide:** (**A**) soft agar colony formation assay was performed in NB-treated SOD2-silenced/-overexpressed HPAC cells. Images were captured at 10 and 40× magnification on day 40; (**B**) the percentage colonies were quantified with HPAC control cells serving as the baseline; (**C**) colony size in µM with a magnification of 40× was quantified and compared between SOD2-altered groups in the presence and absence of NB; (**D**,**E**) Western blot analysis of EMT markers in SOD2-altered groups alone or in combination with NB. Immunofluorescence analysis of E-cadherin expression in (**F**) SOD2-silenced or (**G**) SOD2-overexpressed HPAC cells with or without NB treatment at 100× magnification. Data are shown as mean ± SEM. Experiments (*n* = 3) were repeated three times in triplicates. * *p* < 0.05.

Colony-forming capacities are important events involved in metastatic progression. Soft agar assays were conducted to observe the effects of SOD2 on the colony-forming abilities of HPAC cells. siSOD2 clearly repressed the colony-forming capacity of HPAC cells even after a 40-day incubation period, while ovSOD2 increased the aggressiveness of HPAC cells, which was evident through the number and size of colonies grown (Figure 5A–C). NB-treated cells decreased the anchorage-independent growth potential in both SOD2-silenced and SOD2-overexpressed cells. These results indicate that SOD2 has an important role in PDAC progression and metastasis and highlight the fact that NB inhibits metastasis through an effective suppression of SOD2.

### 3.6. SOD2 Inhibits Epithelial–Mesenchymal Transition of PDAC Cells

Epithelial-to-mesenchymal transition is critical for cell migration during metastasis. We identified increased levels of epithelial marker E-cadherin with the silencing of SOD2, and its expression was more dramatically increased in combination with NB treatment (Figure 5D). The overexpression of SOD2 failed to inhibit NB-induced E-cadherin expression but was able to rescue the expression of many of the mesenchymal markers such as N-cadherin, Zeb1, Slug, Snail, and Twist, which were decreased in response to NB treatment (Figure 5E). Silencing SOD2 reduced EMT markers similar to NB-treated cells. NB treatment in SOD2-silenced cells further increased this inhibitory effect (Figure 5D). The immunofluorescence analysis of E-cadherin showed increased E-cadherin expression in SOD2-silenced NB-treated cells when compared to the NB-alone group (Figure 5F). NB treatment increased E-cadherin expression even in cells overexpressing SOD2 (Figure 5G). Silencing SOD2 in the presence of NB significantly decreased Slug expression levels compared with the NB-alone group (Figure S4A), and a reverse trend was noted in SOD2-overexpressing cells (Figure S4B).

### 3.7. NB-Induced ROS Regulate Apoptosis through SOD2 in PDAC Cells

**Figure 6:**
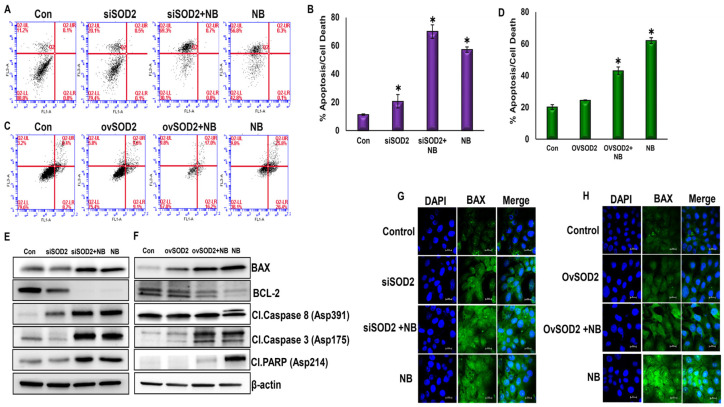
**Nimbolide-induced ROS regulates apoptosis through SOD2 in PDAC cells:** (**A**–**D**) apoptosis was measured in SOD2 genetically altered HPAC cells via flow cytometric analysis using an Annexin-V-FITC Apoptosis Detection Kit I; (**E**,**F**) SOD2-silenced or SOD2-overexpressed HPAC cells in the presence or absence of NB were assessed for key apoptotic biomarkers using Western blot analysis; (**G**,**H**) HPAC pancreatic cancer cells were silenced or overexpressed for the SOD2 gene in the presence and absence of NB to assess the expression levels of proapoptotic BAX via immunofluorescence analysis with 100× magnification. Each bar represents the mean ± SEM of three separate experiments, * *p* < 0.05.

The Annexin-V-FITC and PI-PE apoptosis detection kit was used to analyze the regulation of apoptosis in SOD2-silenced and SOD2-overexpressed HPAC pancreatic cancer cells. Silencing SOD2 increased cell death by 70% in the presence of NB. As expected, NB increased apoptosis by 57.2% compared with the control. Silencing SOD2 without any treatment resulted in 20% cell death (Figure 6A,B). On the other hand, the overexpression of SOD2 decreased apoptosis compared with the NB-alone group. The cell death rate of ovSOD2 cells treated with NB still showed a higher rate than the control but not as high as the NB-treated group (Figure 6C,D). These data suggest that NB-induced ROS regulates apoptosis in PDAC cells through SOD2. The expression of proapoptotic protein BAX was increased in SOD2-silenced cells in the presence of NB, compared with the NB-only treatment group. SOD2 overexpression decreased BAX expression when compared to the NB-alone group. The combination group (siSOD2 + NB) did not show further pronounced effects on other apoptotic markers such as BCL-2, cleaved caspase 3, cleaved caspase 8, and cleaved PARP compared with the NB-alone group (Figure 6E); by contrast, as expected, the overexpression of SOD2 inhibited NB-induced inhibitory effect on all these markers except on cleaved PARP (Figure 6F). Immunofluorescent staining for BAX and cleaved caspase 3 also exhibited similar trends (Figure 6G,H and Figure S5A,B).

### 3.8. Impact of SOD2 Expression on Tumor Growth, Proliferation, Epithelial-to-Mesenchymal Transition, and Apoptosis in Pancreatic Cancer Xenograft Model

**Figure 7:**
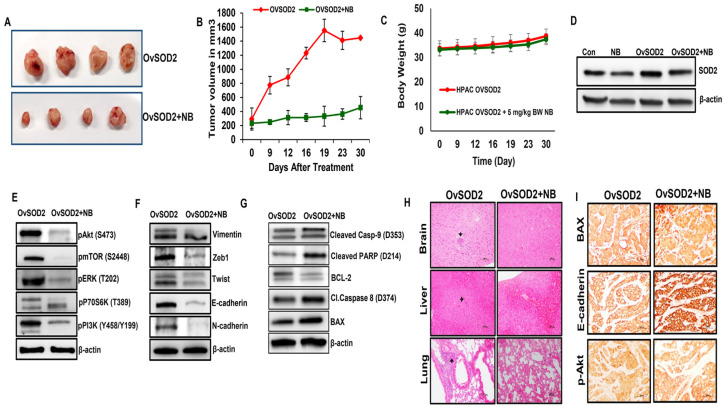
**Nimbolide inhibits pancreatic cancer growth and metastasis through SOD2 inhibition in HPAC xenograft models.** Nude mice bearing xenograft tumors from SOD2-overexpressed HPAC cells were divided into two groups when tumors reached ~100 mm^3^ in size, namely SOD-overexpressed group (ovSOD2) and ovSOD2 mice treated with 5 mg/Kg body weight of NB group; (**A**) representative data demonstrating excised tumor from both ovSOD2 and NB-treated ovSOD2 mice; (**B**) tumor volume and (**C**) body weight were measured after 30 days of treatment with NB; (**D**) Western blot analysis of SOD2 expression from tumor tissues after a month of treatment with NB in animals overexpressing SOD2. Western blot analysis of key markers involved in (**E**) cell proliferation, (**F**) EMT, and (**G**) apoptotic markers from SOD2-overexpressed HPAC xenografts in the presence and absence of NB treatment; (**H**) micrometastasis (indicated with black arrows) were observed in the brain, lung, and liver of ovSOD2 xenograft mice compared with NB-treated ovSOD2 group using hematoxylin-and-eosin staining; (**I**) expression levels of BAX, E-cadherin, and pAkt were measured using IHC staining from NB-treated ovSOD2 HPAC xenografts tissues compared with SOD2-overexpressed control mice. Repeated-measure analysis of variance and Dunnett post hoc test were performed to determine statistical significance.

SOD2-overexpressing HPAC cells (3 × 10^6^) (Figure S5C) were injected into both the flanks of the athymic nude mice. The mice bearing 500 mm^3^ were randomly separated into two groups. From day 10, NB (5 mg/kg BW) was administered twice a week for 4 weeks. Upon the completion of NB treatment, the tumors were surgically excised (Figure 7A). SOD2-overexpressed xenografts without NB treatment expanded their volume by fourfold when compared to the NB-treated group (Figure 7B). The body weights of the mice from both groups were comparable and did not change significantly (Figure 7C). As predicted, SOD2 protein expression in xenograft tissue was significantly higher in ovSOD2 tumors compared with control mice. The obtained data demonstrated NB’s potential to suppress SOD2 to control tumor growth (Figure 7D). Next, the expression levels of cell proliferation markers (pPI3K, pAkt, pmTOR, pERK, and pp70s6K,), EMT markers (Vimentin, Zeb1, Twist, E-cadherin, and N-cadherin), and apoptosis markers (BAX, BCL-2, cleaved caspase 8, cleaved caspase 9, and cleaved PARP) were evaluated using Western blot, and the results were consistent with our in vitro results (Figure 7E–G). Finally, H&E staining demonstrated reduced micrometastasis in the brain, liver, and lung in the NB-treated group compared with untreated SOD2-overexpressed mice (Figure 7H). These in vivo findings confirm the in vitro results supporting the NB’s anticancer effect in pancreatic ductal adenocarcinoma. To validate the relationship between SOD2 and pancreatic cancer development in vivo, immunohistochemistry (IHC) analyses for proliferation (pmTOR), EMT (E-cadherin), and apoptosis marker (cleaved caspase 9) were assessed on xenograft tumor tissues (Figure 7I). IHC results showed similar trends observed in our in vitro studies. E-cadherin and BAX expression increased with NB treatment even in the presence of SOD2 overexpression. The proliferative marker pAkt displayed decreased expression with the NB treatment in SOD2-overexpressing tumor tissues. All these data suggest that NB inhibits pancreatic cancer growth by altering SOD2 levels, resulting in the generation of excessive ROS.

## 4. Discussion

ROS is a double-edged sword, which plays a pivotal role in every step and stage of cancer development. It has been shown to inhibit or promote cancer cell growth based on several factors such as type of chemotherapeutic agent, signaling pathway, type of cancer, and tumor stage [34]. However, the mechanism of action is still poorly understood, and it is crucial to identify specific cellular targets for it to be used as an effective druggable target. In our previous study, we showed that NB’s anticancer effects were through increased ROS generation [28]. Here, we show that NB induces ROS by silencing SOD2 and inhibiting the PI3K/Akt signaling pathway.

The SOD family of enzymes (SOD1, SOD2, and SOD3) are recognized as the principal oxidative stress defense mechanism regulators as they efficiently combat ROS [35]. We demonstrated that NB induced cell death mainly through mitochondrial-mediated apoptosis [28]. Through IHC analysis we found that SOD2 isoform is overexpressed in human pancreatic tumor tissues and pancreatic cancer cell lines, and SOD2 has a higher expression than other isoforms of SOD. Our data demonstrate that an elevated expression level of SOD2 positively correlates with increasing tumor grade and decreased overall survival and recurrence-free survival time in pancreatic cancer patients. Previously, SOD2 has been described as having a dichotomous nature as a suppressor and promotor of tumors [36]. Our data indicate that SOD2 acts as a tumor promotor in pancreatic cancer patients. Silencing SOD2 diminished the proliferative capacity of pancreatic cancer cells, which was further reduced by NB treatment. The overexpression of scavenger SOD2 was able to normalize superoxide levels while increasing hydrogen peroxide levels in favor of cancer cell growth, which resulted in decreasing the effect of NB. Conversely, silencing SOD2 reduced the levels of hydrogen peroxide and the accumulation of highly reactive superoxide molecules. Silencing SOD2 made PDAC cells more sensitive to NB. SOD2 inhibition in conjunction with NB increased ROS levels and inhibited PI3K/Akt signaling, while the inhibition of ROS increased PDAC cell proliferation even in the presence of NB, indicating a possibility for feedback regulation between ROS and PI3K/Akt signaling in NB-treated pancreatic cancer cells. NB decreased the activity of SOD2, which led to increasing ROS levels in the mitochondria tipping the redox system off their physiologic balance. Cells are not able to tolerate high ROS levels, resulting in their deterioration. This is further confirmed by our data, indicating that the overexpression of SOD2 counteracts the effect of NB. It has been shown that NB leads to cell cycle arrest and induces cell apoptosis via the activation of caspases 3, 8, and 9 [37].

In our study, we observed that ROS levels decreased when SOD2 was overexpressed, resulting in increased aggressiveness of PDAC cells. We also found that NB decreases SOD2 expression and negatively impacts the PI3K/Akt/mTOR pathway, leading to the inhibition of growth in pancreatic cancer cells. This pathway is crucial for the growth and survival of several cancer cell types [38,39]. The silencing of SOD2 inhibited PDAC proliferation due to the increased accumulation of ROS, thereby inhibiting the active forms of key proliferative markers Akt, PI3K, ERK, mTOR, and P70S6K. The overexpression of SOD2 in the presence of NB indicated a strong possibility for the regulation of ROS and PI3K/Akt signaling. Upon the assessment of ROS, it was found that the induction of ROS by NB is an early event that may lead to the inhibition of PI3K/Akt signaling in pancreatic cancer cells. To explore the potential mechanism of action of NB, we investigated if the inhibition of PI3K/Akt signaling by NB regulates ROS generation or if NB-induced ROS inactivates Akt signaling. Our data demonstrated that both regulated each other, suggesting a strong possibility for feedback regulation between ROS and PI3K/Akt signaling in NB-treated pancreatic cancer cells.

SOD2 inhibition and NB were effective in suppressing the aggressive and metastatic characteristics of pancreatic cells, as evidenced by lower levels of migration, invasion, and colony-forming capabilities. The process of epithelial-to-mesenchymal transition is a hallmark of aggressive and highly invasive cancers [40]. During EMT, epithelial properties are lost or reduced, while mesenchymal traits are gained, resulting in a loss of cell-to-cell adhesion that promotes the invasion and eventual induction of metastasis [41]. Furthermore, ROS production has been shown to alter the activity of pivotal enzymes for cell dynamics, causing the reorganization of actin cytoskeleton, adhesion, and cell migration, which affects EMT dynamics in the cells [42,43]. Our data demonstrate that SOD2 silencing inhibits EMT by decreasing the expression of Snail, Twist, Slug, Zeb1, and N-cadherin while increasing the expression of epithelial marker E-cadherin. The antimetastatic effect of NB could be attributed to SOD2 inhibition and an increase in ROS production that regulates the EMT process. Together, our data show that NB inhibits SOD2 and increases ROS to inhibit proliferation, migration, invasion, and colony formation in pancreatic cancer cells. NB also influences apoptosis through SOD2. Our data demonstrated that silencing SOD2 increased the expression of proapoptotic markers (BAX, cleaved caspases 3 and 8, and cleaved PARP), while the expression of antiapoptotic marker BCL-2 was decreased. A reverse of this effect was observed when SOD2 was overexpressed, indicating that the NB-induced alteration of SOD2 expression plays a vital role in PDAC apoptosis.

Xenografted PDAC cells overexpressing SOD2 exhibited rapid tumor growth in vivo. NB treatment was highly effective in inhibiting the aggressive tumor growth of SOD2-overexpressing PDAC xenografts. We observed that NB suppressed SOD2 to reduce tumor growth by targeting cell proliferation, EMT, apoptosis, and PI3K/Akt signaling in SOD2-overexpressing PDAC xenograft tumors. Similar to our in vitro results, we also observed that NB suppressed key markers of PI3K/Akt signaling pathway, EMT, and apoptosis. Interestingly, NB also reduced the micrometastasis of SOD2-overexpressing PDAC cells in the brain, liver, and lung. These data further suggest that a possible mechanism through which NB inhibits PDAC growth and progression could be by decreasing the expression of SOD2, resulting in increased accumulation of ROS. 

## 5. Conclusions

In conclusion, our data delineate a potential anticancer mechanism of action of NB, which is NB induces ROS generation mainly through the inhibition of SOD2, which inhibits proliferation via PI3K/Akt signaling pathway and metastasis while increasing apoptosis in pancreatic cancer cells. Further exploration of NB’s concrete mechanism with regard to pancreatic cancer is warranted and could provide an effective treatment option for PDACs.

## Data Availability

Data are contained within the article and Supplementary Materials.

## Supplementary Materials

The following supporting information can be downloaded at: https://www.mdpi.com/article/10.3390/antiox12101791/s1, Figure S1: Quantitative analysis of SOD2 expression; Figure S2: Changes in phosphoproteome by nimbolide influences various signaling pathways; Figure S3: Silencing SOD2 enhances the impact of nimbolide on pancreatic cancer cells by decreasing the expression of proliferative marker pmTOR; Figure S4: Silencing SOD2 in the presence of nimbolide attenuates the expression levels of Slug; Figure S5: Nimbolide increases the expression of cleaved caspase 3 apoptotic marker in pancreatic cancer cells.
