# Supplementary material for: Nimbolide Inhibits SOD2 to Control Pancreatic Ductal Adenocarcinoma Growth and Metastasis
Source: Antioxidants (Basel). 2023 Sep 22;12(10):1791. doi: 10.3390/antiox12101791 (PMC10604165; doi:10.3390/antiox12101791)
Supplement: Supplementary file 1 [file antioxidants-12-01791-s001.zip › antioxidants-2603827-supplementary.pdf]

Supplementary Material:

Supplementary Materials and Methods:

Cell culture:

HPAC, PANC-1, BxPC-3 and AsPC-1 cells were purchased from American Type Culture Collection (ATCC) and cultured in RPMI-1640 media (Gibco, Life Technologies, 22400-089), supplemented with 10% Fetal Bovine Serum (FBS; Gibco, Life Technologies, 10437036), 100 ug/mL of penicillin, and 100 µg/mL of streptomycin (Pen/Strep, Gibco, Life Technologies, 15070-063). MIAPaCa-2 were cultured using DMEM media supplemented with 10% FBS, 2.5% horse serum, 100 units/mL of penicillin, and 200 mM glutamine. To culture Capan-1 cells, Iscove's modified Dulbecco's medium supplemented with 20% FBS was used. Capan-2 cells were maintained in ATCC-formulated McCoy's 5a medium added with 10% FBS. hTERT HPNE cells were cultured in 75% DMEM and 25% M3 base medium supplemented with 5% FBS, 10 ng/ml human recombinant EGF, 5.5 mM D-glucose, and 750 ng/ml puromycin. The cell lines were passaged for no more than 6 months. Nimbolide (NB) was purchased from BioVision, Inc. (1891-5).

Immunohistochemistry:

Tumor sections were deparaffinized by heating at 58oC for 2h and followed by xylene treatments. The tissue sections were rehydrated using serially decreasing ethanol baths and finally in water. Antigen retrieval with triology (Cell Marque, Rocklin,CA) was performed and the tissue sections were blocked and then probed with antibodies for overnight at 4°C. After washing, slides were incubated in signal amplifier (UltraMarque polyscan HRP label, Cell Marque, Rocklin, CA) at room temperature for 1 hour. Protein expression in tissue sections were visualized using DAB and

counterstained with hematoxylin. Mounting media was used to seal the slides (Surgipath Medical Industries, Richmond, IL, USA), and images were captured with a Nikon Microscope– ECLIPSE 50i at 10, 20 and 40X magnification.

Immunofluorescence analysis:

Pancreatic cancer cells were seeded in 8-well chamber slides at a density of 10,000 cells/well. As previously described (29976975), cells were transfected with either SOD2 siRNA (siSOD2) or SOD2 plasmid DNA (ov SOD2) for 48h hours. Post transfection, NB was treated either alone or in combination with siSOD2 or ovSOD2 transfected cells for 24h. The cells were then fixed with 100% methanol/acetone and permeabilized using 0.2% Triton-X in PBS for 20 min. After blocking with 5% BSA, the cells were incubated with the following antibodies: SOD2 (13141), p-Akt (4060), p-mTOR (5536), E-cadherin (3195), Slug (9585), and BAX (2772), purchased from Cell Signaling Technology (Danvers, MA, USA); Cleaved Caspase 3 (c8487), purchased from Sigma-Aldrich (St. Louis, MO, USA). Secondary antibody Alexa fluor 488 (green) (Invitrogen, Waltham, MA, USA) was used to detect the above mentioned proteins. Images were captured using a Nikon Eclipse Ti laser scanning confocal microscope.

Immunoblotting:

Equal amounts of protein from various experimental groups were loaded and electrophoresed using sodium dodecyl sulfate-polyacrylamide gel (Bio-Rad Mini-Protean TGX Precast Gel). Proteins were then transferred onto polyvinylidene difluoride membranes (Millipore Immobilon-P transfer, IPVH00010). The membranes were probed with the following primary antibodies: SOD2, pAkt, pMTOR, pP70 S6K, pPI3K, Vimentin, E-Cadherin, Cleaved Caspase 8, Cleaved Caspase 9, BAX, N-Cadherin, Snail purchased from Cell Signaling Technology (Danvers, MA,

USA); ß-actin (A1978) purchased from Sigma-Aldrich (St. Louis, MO, USA); Zeb-1 and pERK purchased from Santa Cruz Biotechnology (Santa Cruz, CA, USA); Twist, purchased from Abcam (Cambridge, UK). Nonspecific binding of primary antibody was blocked with 5% bovine serum albumin (BSA; Sigma, A4503). Blots were then incubated with primary antibodies (see supplementary methods), Blots were then washed with TBST and incubated for 1 hour at room temperature in horseradish peroxidase-coupled secondary antibodies, including: Rabbit, anti-mouse (SC-358914, 1:1000) and Goat anti-Rabbit (SC-2004, 1:1000), purchased from Santa Cruz Biotechnology (Santa Cruz, CA, USA). Protein of interest were detected with either SuperSignal West Pico PLUS Chemiluminescent Substrate (Thermo Fisher Scientific, Inc., 34579) or SuperSignal West Femto Maximum Sensitivity Substrate (Thermo Fisher Scientific, Inc., 34096). Blots were imaged using Imager GE Las-4000 (GE Healthcare Life Sciences, Chicago, IL, USA). Densitometric analysis was performed using Image Studio Lite (Ver5.2.5) (LI-COR Biosciences, Lincoln, NE, USA). The relative protein expression are represented as the density ratio versus ß-actin.

Transwell migration and matrigel invasion assay:

Transwell inserts with 8.0 µm pore polycarbonate membranes were purchased from Corning Incorporated (Costar; Corning, NY, USA). HPAC cells were seeded into 6-well plates at a density of 5x105 cells/well. After 24 hours, cells were transfected with siSOD2, or SOD2 pcDNA using the previously mentioned corresponding transfection reagents. After 48 hours, the transfection complex was removed and the cells were treated with 3µM NB. Following 24 hour treatment, cells were trypsinized, counted, and reseeded in serum-free media into Transwell chambers at a density of 2.5x103 cells per insert for migration and 1.5x104 for invasion. For invasion assay transwell inserts were coated with 1mg/ml of matrigel. Complete media was added into bottom of the wells

as a chemoattractant. Non-transfected and untreated cells were also used as controls. After a 48 hour incubation at 37°C, migrated/invaded cells were fixed with 5% formalin (VWR, 16004-128) and stained using 0.2% Crystal Violet (Sigma, C3886) for 1 hour at room temperature. Inserts were washed with PBS and non-migrating cells were removed with cotton swabs. Images were captured and counted in randomly selected fields of view using a Nikon Eclipse TS 100 microscope at varying magnifications.

Soft agar colony-formation assay:

Colony formation assay as performed in siSOD2, or ovSOD2 transfected HPAC cells were treated with 5 μM NB. After 24 hours, cells were reseeded at a density of $1.5x10^5$ cells into 60 mm petri dishes with a top layer of 0.7% agarose (Agarose Ultrapure, Invitrogen,16500-100) and a bottom layer of 1% agar (Agar Difco Nutrient, Becton Dickinson, DF001-17-0). Non-transfected and untreated cells were used as controls. Cells were maintained for up to 45 days and then stained with 0.2% crystal violet. Images of cell colonies were captured using a Nikon SMZ 1500 microscope at 10 and 40X magnifications.

Wound healing assay:

Wound healing assay was performed in SOD2-silenced and SOD2-overexpressing HPAC cells with or without NB treatment. Transfected cells were seeded in 6-well plates at a density of 1.2×10^6 cells/well and maintained for 96 h. A scratch was created with a sterile pipette tip once the cells reached monolayer confluency. The distance migrated by the cells were captured and calculated at 2 h intervals for 96 h using the Nikon Biostation CT. NIS-Element AR software was used for analysis.

Apoptosis analysis and flow cytometry:

Apoptosis was studied using Annexin V-FITC Apoptosis Detection Kit (556547) according to the manufacturer's instructions (BD Biosciences). Cells were seeded in 6-well plates at a density of 5×10^5 cells/well and were treated with 5 μM NB for 24 h or transfected with SOD2 plasmid or siRNA for 48 h. Apoptosis was studied using FACS Accuri C6 flow cytometer (San Jose, CA, USA).

Phospho-explorer antibody array:

HPAC at a density of 2.5×10^5 cells/well were seeded on a six well plate to perform ELISA based phospho explorer antibody array (Full Moon Biosystems, Pex100). Following the treatment with NB at a concentration of 5μM for 24 h, cells were harvested to extract protein lysates and then purified through glass bead columns as specified in the manufacturer's instructions. Protein concentrations were measured through UV absorbance spectroscopy (Nanodrop, Thermofisher Scientific). Array slides were then blocked and incubated with the protein coupling mix at room temperature which consist of over 1,300 phosphorylated and unphosphorylated proteins. Slides were then submerged into a 100 x 15 mm Petri Dish with Cy3-Streptavidin solution and incubated for 20 min at room temperature on an orbital shaker rotating at 35 rpm to ensure even coating of the slides. Slides were washed with 1X wash solution followed by 10 washes with MilliQ grade water. Array slides were dried via centrifugation at 13000 x g for 10 minutes to detect fluorescent intensity. The slides were scanned using an Axon GenePix Array Scanner (Molecular Devices) and the intensity values were normalized with internal controls.

Silencing SOD2 in pancreatic cell line:

Three different SOD2 siRNA subtypes were obtained from OriGene (Cat# SR321855, Rockville, MD, USA) with different sequences were used to silence SOD2. Mirus bio TransIT siQUEST (Cat#MIR2110, Marietta, GA) transfection reagent was used for transfection. A scramble siRNA sequence was used as a negative control for non-sequence-specific effects. Among the three subtypes of SOD2 siRNA, siRNA 'A' showed maximum efficacy in silencing SOD2. Following the 48 hour transfection, cells were processed for further experiments.

SOD2 siRNA subtypes sequences:

SR304519A – rGrGrGrArGrArArUrGrUrArArCrUrGrArArArGrArUrArCAT

SR304519B – rCrCrArCrUrGrCrArArGrGrArArCrArArCrArGrGrCrCrUTA

SR304519C – rArGrUrArArArCrCrArCrGrArUrCrGrUrUrArUrGrCrUrGAG

Overexpression of SOD2 in pancreatic cell line:

A plasmid overexpressing SOD2 (ovSOD2) gene was obtained from OriGene (CW303931, Rockville, MD, USA). HPAC cells were seeded in 6-well plates at a density of $5x10^5$ cells/well and transfected with SOD2 plasmid DNA at a concentration of 10µg/µL using Mirus 2020 (Cat#MIR5400, Marietta, GA) transfection reagent. An empty plasmid vector was used as a control. After 48 hours of transfection, green fluorescence analysis was performed using a FLoidCell Imaging Station (Life Technologies, Thermo Fisher Scientific, Inc.) to determine the efficacy of transfection for further analysis.

Cell proliferation using MTS assay:

HPAC cells were seeded in a 96-well plate at a density of $5x10^3$ cells per well in triplicates. Cell proliferation was determined by using MTS assay. HPAC cells were exposed to NB at varying doses for 24 h. According to the manufacturer's protocol, MTS reagent was added into each well and incubated 4h. Optical density was measured using a BMG Labtech CLARIOstar microplate reader at 490 nm absorbance.

ROS generation assay:

Intracellular ROS generation was assessed using the OxiSelect Intracellular ROS assay kit as described earlier purchased from Cell BioLabs, Inc. (STA-342). HPAC cells were seeded in a 96-well plate at a density of $2x10^4$ cells per well. After 24 hours, cells were treated with Wortmannin, LY294002, NAC for 24h or transfected with SOD2 siRNA, or SOD2 pcDNA for 48h. Then, cells were washed with PBS for three times, then incubated with 2',7'-dichlorodihydrofluorescin diacetate (DCFH-DA) for 45 minutes at 37°C in the dark. DCFH-DA was then removed and cells were treated with 5 μM NB. After 20 minutes, 1x cell lysis buffer was added and mixed thoroughly for 5 minutes. Cell lysate from each replicate were transferred into a black bottom fluorimetric 96-well plate for fluorescence measurement using a BMG LabTech fluorescence plate reader at 480 nm excitation and 530 nm emission. Data are expressed as the amount of ROS relative to non-transfected and untreated control groups.

Supplementary Figures and **Figure Legends:**

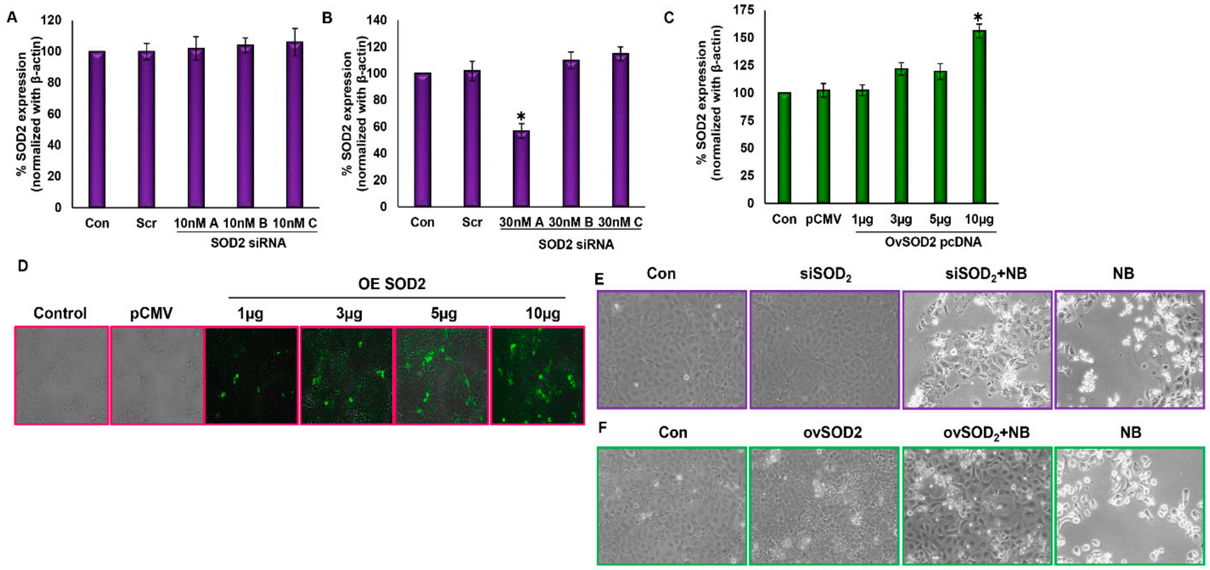

Figure. S1: Quantitative analysis of SOD2 expression.

A&B. Densitometric analysis of Western blot data represented in Figure 2a using different subtypes of siRNA ranging from 10 to 30 nm in HPAC pancreatic cancer cells for 48h. **C.** Densitometric analysis of Western blot data represented in Figure 2c using SOD2 overexpressing plasmid cDNA raging frim 1 to10 µg HPAC pancreatic cancer cells for 48h. **D.** GFP reporter gene expression from non-transfected control cells, pCMV and SOD2 over expressed HPAC pancreatic cancer cells with various concentration of GFP tagged plasmid pcDNA (1, 3, 5, and 10 µg). **E&F.** Morphological changes of SOD2 silenced/overexpressed HPAC pancreatic cancer cells with and without NB respectively.

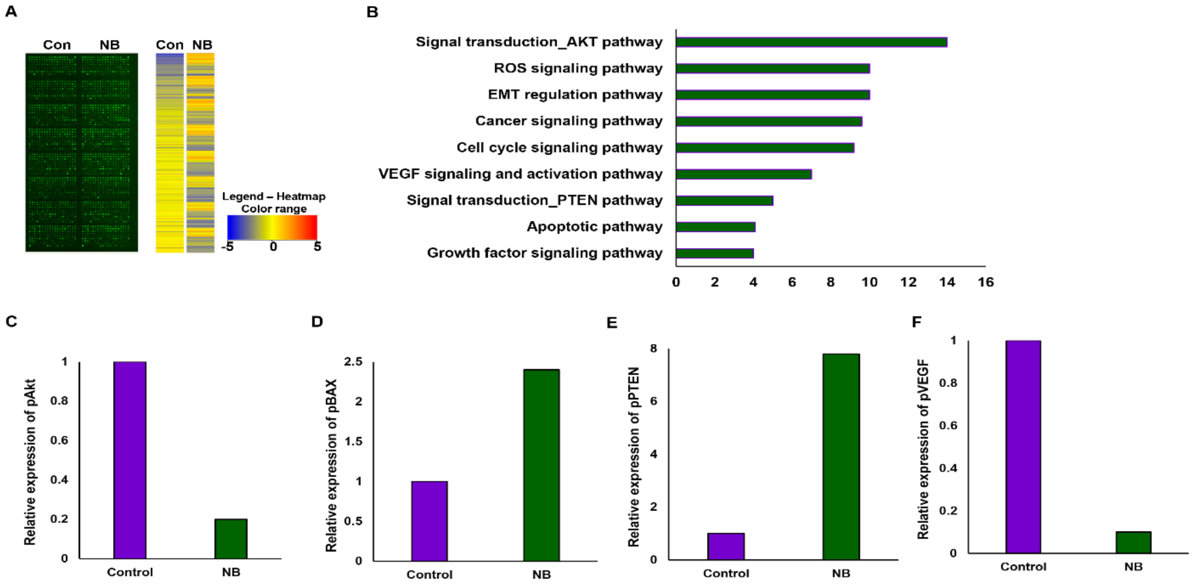

Figure. S2: Changes in phosphoproteome by nimbolide influences various signaling pathways.

A. Heatmap analysis from Phospho-explorer antibody array data were represented to show the differential expression of proteins and their phosphorylation status by NB compared to control HPAC cells. **B.** Nimbolide treatment alters key biological pathways in pancreatic cancer cells. Nimbolide influenced key signaling protein expression levels such as **C.** pAkt, **D.** pBAX, **E.** pPTEN **F.** pVEGF within the total phosphoproteome of pancreatic cancer cells.

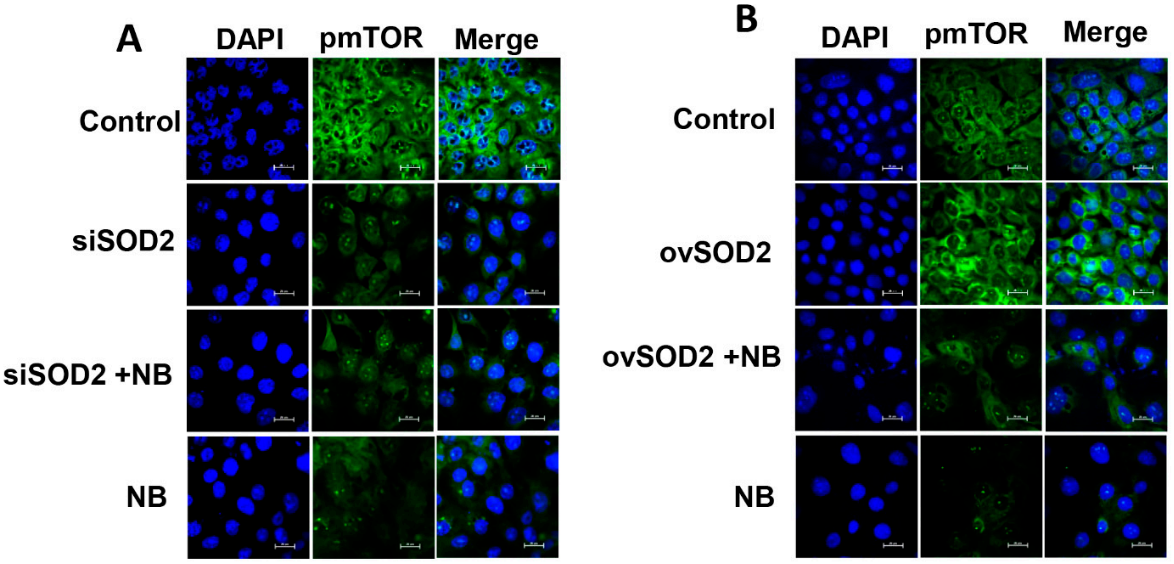

Figure. 3: Silencing SOD2 enhances the impact of nimbolide on pancreatic cancer cells by decreasing the expression of proliferative marker pmTOR.

A. Proliferation marker, pmTOR expression levels were recorded through immunofluorescence in HPAC pancreatic cancer cells after 24 h treatment with NB in siSOD2 cells. **B.** pmTOR expression were analyzed in SOD2 overexpressed HPAC cells with or without NB.

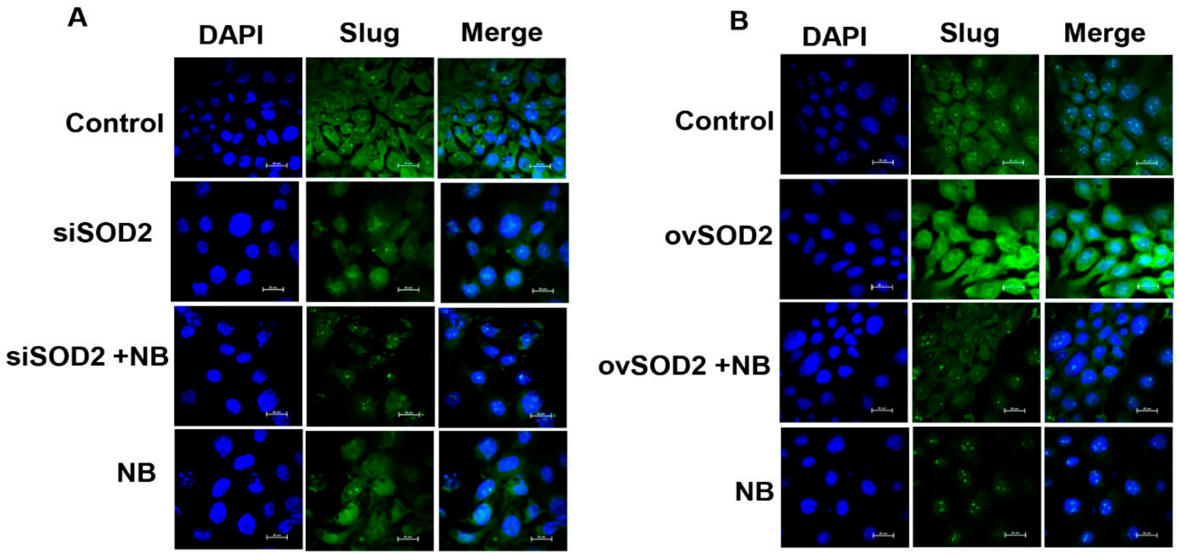

Figure. S4: Silencing SOD2 in the presence of nimbolide attenuates the expression levels of slug.

A. EMT marker expression, Slug was recorded through immunofluorescence in SOD2 silenced HPAC pancreatic cancer cells following treatment with NB. **B.** Slug expression were analyzed in SOD2 overexpressed HPAC cells with or without NB.

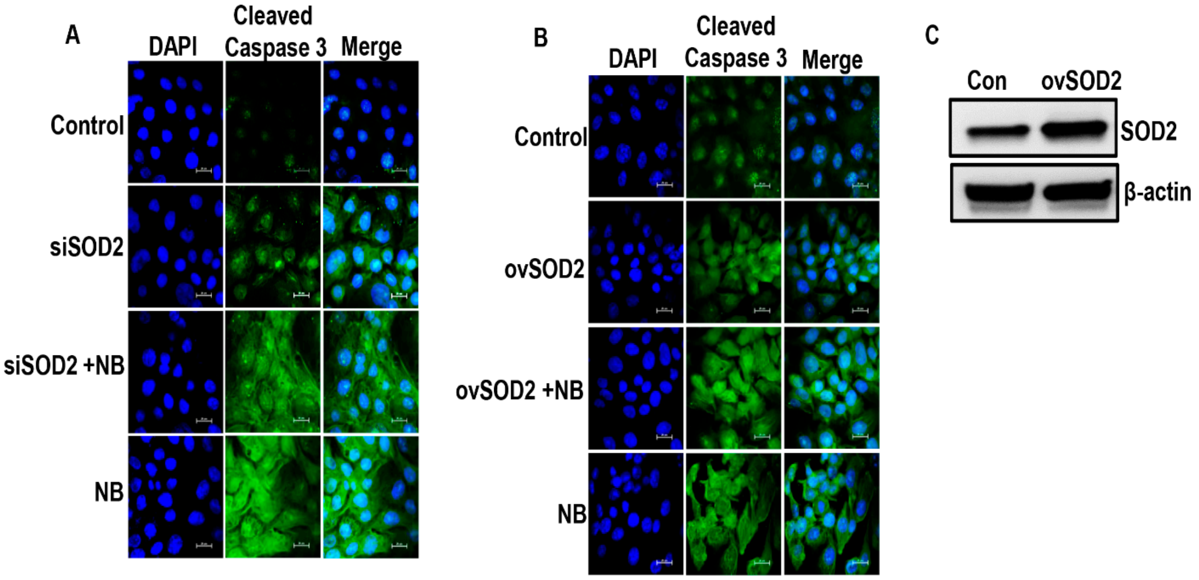

Figure. S5: Nimbolide increases the expression of cleaved caspase 3 apoptotic marker in pancreatic cancer cells.

A. Cleaved caspase 3 apoptotic marker expression was recorded through immunofluorescence in SOD2 silenced HPAC pancreatic cancer cells following treatment with NB. **B.** Cleaved caspase 3 expression were analyzed in SOD2 overexpressed HPAC cells with or without NB. **C.** Western blot analysis of SOD2 protein expression in ovSOD2 HPAC cells.
